# Endocrine Disrupting Compounds – do we need to consider thyroid hormones? Mechanism-based testing strategy using in vitro approaches for identification of thyroid hormone disrupting chemicals

**DOI:** 10.1186/1756-6614-6-S2-A20

**Published:** 2013-04-05

**Authors:** Arno C Gutleb

**Affiliations:** 1 Centre de Recherche Public – Gabriel Lippmann, Luxembourg

## 

The thyroid hormone (TH) system is involved in many important physiological processes, including regulation of energy metabolism, growth and differentiation, development and maintenance of brain function, thermo-regulation, osmo-regulation, and axis of regulation of other endocrine systems, sexual behaviour and fertility, cardiovascular function. Therefore, concern about TH disruption (THD) has resulted in strategies being developed to identify THD chemicals (THDC). Information on potential of chemicals causing THD is typically derived from animal studies. However, for most chemicals, this information is often limited or even unavailable. It is also unlikely that animal experiments will be performed for all THD relevant chemicals in the near future for ethical, financial and practical reasons. In addition, typical animal experiments often do not provide information on the mechanism of action of THDC, making it harder to extrapolate results across species. Relevant effects may not be identified in animal studies when the effects are delayed, life stage specific, not assessed by the experimental paradigm (e.g., behaviour) or only occur when an organism has to adapt to environmental factors by modulating TH levels. Therefore, in vitro and in silico alternatives to identify THDC and quantify their potency are needed. THDC have many potential mechanisms of action, including altered hormone production, transport, metabolism, receptor activation and disruption of several feed-back mechanisms. In vitro assays are available for many of these endpoints, and the application of modern ‘-omics’ technologies, applicable for in vivo studies can help to reveal relevant and possibly new endpoints for inclusion in a targeted THDC in vitro test battery. Within the framework of the ASAT initiative (Assuring Safety without Animal Testing), an international group consisting of experts in the areas of thyroid endocrinology, toxicology of endocrine disruption, neurotoxicology, high-throughput screening, computational biology, and regulatory affairs has reviewed the state of science [1] for (1) known mechanisms for THD plus examples of THDC; (2) in vitro THD tests currently available or under development related to these mechanisms; and (3) in silico methods for estimating the blood levels of THDC. Based on this scientific review, the panel recommends a battery of test methods to be able to classify chemicals as of less or high concern for further hazard and risk assessment for THD.
